# The effects of yoga on mental health in school-aged children: A Systematic Review and Narrative Synthesis of Randomised Control Trials

**DOI:** 10.1177/13591045221136016

**Published:** 2022-10-27

**Authors:** Kirti Khunti, Sadie Boniface, Emma Norris, Cesar M De Oliveira, Nicola Shelton

**Affiliations:** 1 154305 University College London (UCL), London, UK; 2 Kings College London, London, UK; 3 3890 Brunel University, London, UK

**Keywords:** Yoga, mindfulness, school, children, adolescents, mental health

## Abstract

It is becoming increasingly common for frontline clinicians to see children and teenagers struggle with their mental health. Since mental health issues have increased over the past ten years in the UK, they are now the leading cause of disability and cost the British economy £105 billion annually. The review discusses the evidence base underpinning the effect of yoga on children’s mental health and summarises the results of 21 research papers. The Cumulative Index to Nursing and Allied Health Literature, PsycINFO, ERIC, Web of Science, PubMed, Medline and Cochrane Library were searched through Ovid from January 2008 until May 2022. The keywords 'yoga OR mindfulness – AND school AND children OR child OR youth OR adolescent' were used. The search was limited to studies in the English language. The quality of each study was rated against Version 2 of the Cochrane risk-of-bias tool for randomised control trials and a set of inclusion and exclusion criteria. The evidence for yoga therapies in children is encouraging, although studies include methodological flaws such as small sample sizes and sparse information on interventions. This review has highlighted that yoga interventions may be implemented in schools as a preventative and therapeutic measure for mental health issues.

## Background

The National Health Service (NHS) reported that 10.8% of children aged between 5 and 16 in England suffered at least one mental disorder in 2017. The incidence has since risen to 16.0% in July 2020 across age, gender, and ethnic groups during the Covid-19 pandemic (Newlove-Delgado et al., 2021). With the rise of mental health problems in the United Kingdom (U.K.) in the last decade, it is now the single most significant cause of disability and costs the U.K. economy £105 billion a year, which is almost the entire cost of the NHS (NHS, 2018). Mental health problems in children in the U.K. are seen in children as young as 2 years, and each month almost 50,000 children and adolescents are referred for mental health interventions or treatment by the NHS (NHS, 2018). It is a problem worldwide; for example, according to the World Health Organization (WHO, 2020b), approximately one million school children aged between 11-15 years have some form of mental health disorder, such as feeling low and anxious. In particular, increasing numbers of children across the European Region have reported poor mental health. The WHO report claims that children are more prone to mental health conditions as they age. Some causes of mental health in children are multiple physical, emotional and social changes, such as exposure to poverty, abuse, or violence leading to emotional disorders such as depression or anxiety, irritability, frustration or anger. Symptoms can overlap across more than one emotional disorder with rapid and unexpected changes in mood and emotional outbursts; adolescents may also develop emotion-related physical symptoms such as headaches or stomach aches (WHO, 2020a). Stress experienced in childhood includes sexual, physical or emotional abuse, neglect, parental mental illness, parental divorce or separation, exposure to violence, substance abuse, and low socioeconomic status.

Stress during child development can lead to cognitive consequences in adult life (Britton et al., 2014). Mendelson et al. (2010) highlighted that some of the significant risk behaviours in children include teenage pregnancy/parenting, unsafe sex, crime and violence, drug/alcohol use and abuse, underachievement, or school failure. Many adolescents experience symptoms of anxiety and depression that can lead to adverse outcomes on social and family functioning, as well as demographic factors, such as sex, ethnic group, family functioning, parental mental health, qualification status of the parent, marital status of the parent, family type (Eva and Thayer, 2017).

It is becoming increasingly common for frontline clinicians to see children and teenagers struggle with everyday pressures such as dealing with parental mental health, intense pressure at school, bullying and being bombarded by social media with what is normal. These are the biggest drivers of mental health in children.

However, studies suggest that resilience, for example, managing and coping with stress, can decrease the negative consequences of trauma (Ortiz and Sibinga, 2017). Therefore, the pre-adolescent period is an opportunity for interventions to help prevent the development of poor health outcomes in later adulthood, such as comorbid metabolic and cardiovascular dysfunction and mental health.

The Healthy Child Programme by Public Health England (PHE) suggests that early intervention is paramount for children, especially those living in social difficulties impeding child development (PHE, 2018). The autonomic nervous, endocrine, and immune systems are connected to the brain and body; they work together to facilitate adaptation to stress. Young children and adolescents from disadvantaged communities who experience early and chronic life stressors may affect brain development, this can negatively influence cognitive function and emotional regulation, and may in turn increase the risk of adverse emotional and behavioural outcomes (Dariotis et al., 2016a).

Yoga originated in India. Yoga comes from the Sanskrit word ‘Yuj’, which means *“union of the individual consciousness or soul with the Universal Consciousness or Spirit”* (Perfect and Smith, 2016). Yoga is a practical philosophy; it aims to unite the body, mind and spirit for health and fulfilment, leading to happiness and well-being (Bhavanani, 2011). The first mention of yoga was documented in ancient Hindu scripture and written in 2000 BCE (White, 2009), though yoga is as old as civilisation itself (Wallace and Benson, 1972). Yoga is made up of 3 key intervention components: physical activity (‘asanas’), breathing techniques (‘pranayama’), and mindfulness meditation (Tamilselvi and Mala, 2016).

Mental health may be addressed by introducing yoga interventions as a prevention and treatment solution (Tamilselvi and Mala, 2016). Kim et al. (2016) study found that the practice of a 12-week school-based yoga project showed positive body image in children through controlled breathing, meditation, and calming the mind. However, some educators in their study raised concerns about the lack of confidence in teaching yoga to students. They do not know how to instruct or lead yoga sessions or do not have the time because of tight curriculum schedules (Kim et al., 2016).

Despite yoga’s popularity in some schools, it is not widely used and may be due to the perception of yoga as associated with Hinduism (White, 2009). Therefore, it is crucial to share evidence-based research on the effects of teaching yoga to children in schools and the community without associating it with Hinduism. One way this may be addressed is through meetings, newsletters, or conversations with parents. In White’s (2009) study, schools changed the terminology from pranayama to “bunny breathing” or meditation to “time in.” Chaya et al. (2012) yoga intervention was modified for the children; for example, children were asked to close their eyes during deep inhalations and exhalations and count 20–0 backwards.

Yoga is accepted as a holistic system of practices that includes many techniques, such as physical postures, various breathing exercises, and relaxation techniques (Khalsa and Butzer, 2016), and have shown promise in improving children’s physical (Kongkaew et al., 2018) and mental health (Miller et al., 2020). Studies have attracted interest in developing and applying meditation and yoga-based interventions in schools worldwide (Khalsa and Butzer, 2016). Some researched benefits of practising yoga are higher energy levels, fine motor coordination, muscle tone, flexibility, postural alignment, and cardiovascular fitness (Felver et al., 2020). Also, yoga requires limited space and no equipment; it is easy to learn and has been accepted worldwide (Mehta et al., 2012), and appeared in the U.K. in the early 1970s; approximately 500,000 people practise yoga each week in the U.K. (Wood, 2020).

Yoga with children can allow them to redirect energy positively, helping them calm their minds and bodies, especially during anxiety periods. It may be helpful for those who can be destructive and aggressive. Yoga may increase a child’s well-being, enhance self-worth, and promote fewer negative behaviours. The implementation of yoga in class is fun, easy, and cost-effective, according to Khalsa and Butzer’s (2016) review.

Wolff and Stapp (2019) argued that teachers have an invaluable role in implementing yoga in schools. They found that the breathing techniques acquired during yoga increased the ability to self-regulate and improve attention. In a pilot study, Butzer et al. (2015) found that ten weeks of yoga intervention on children aged between 7-9 years old showed statistically significant cortisol concentration changes, social interaction, attention span, stress coping, confidence, time on task, academic performance, and improved mood.

This systematic and narrative review aimed to examine the effect of yoga on children’s mental health.

## Methods

A systematic and narrative review was conducted, which followed the PRISMA reporting guidelines (Page et al., 2021). PROSPERO was searched for ongoing or recently completed systematic reviews to avoid duplication of systematic reviews. The systematic review protocol was prospectively registered on PROSPERO (CRD42020171943). The systematic review included only RCT studies evaluating intervention processes. This systematic review aimed to compare the effectiveness of different interventions and estimate how much difference the intervention is likely to make if applied in practice. The findings of the included studies were brought together in a narrative synthesis.

### Search strategy

A search was performed for published papers using the Cumulative Index to Nursing and Allied Health Literature (CINAHL), PsycINFO, ERIC, Web of Science, PubMed, Medline and Cochrane Library searched through Ovid. Results were searched from January 2008 until May 2022. Searching for papers between 2008 ensured that the maximum number of papers were found. Preliminary searches found that most RCTs were carried out between 2010 to 2021, and this may be due to the increased awareness of child mental health and/or yoga in high income countries, primarily as most papers found were based on studies carried out in the USA.

The keywords ‘Yoga OR mindfulness – AND school AND children OR child OR youth OR adolescent’ were used. The search was limited to studies in the English language. However, the word mindfulness was used to capture the most research articles possible, as some studies had used mindfulness as a secondary outcome. No mental health search terms were used because the study outcomes were diversely measured and used various languages.

Studies were selected in line with the predefined PICOS:

**Population:** Primary and secondary school children aged 5-16, anywhere.

**Intervention:** School-based yoga intervention measuring the effects of yoga on mental health.

**Comparator:** Any, including physical education (P.E.) or education as usual.

**Outcomes:** Depression (low mood), self-esteem, stress or anxiety outcomes as either a primary or secondary outcome.

**Study design:** – Randomised Control Trials (RCTs).

**Figure 1.**
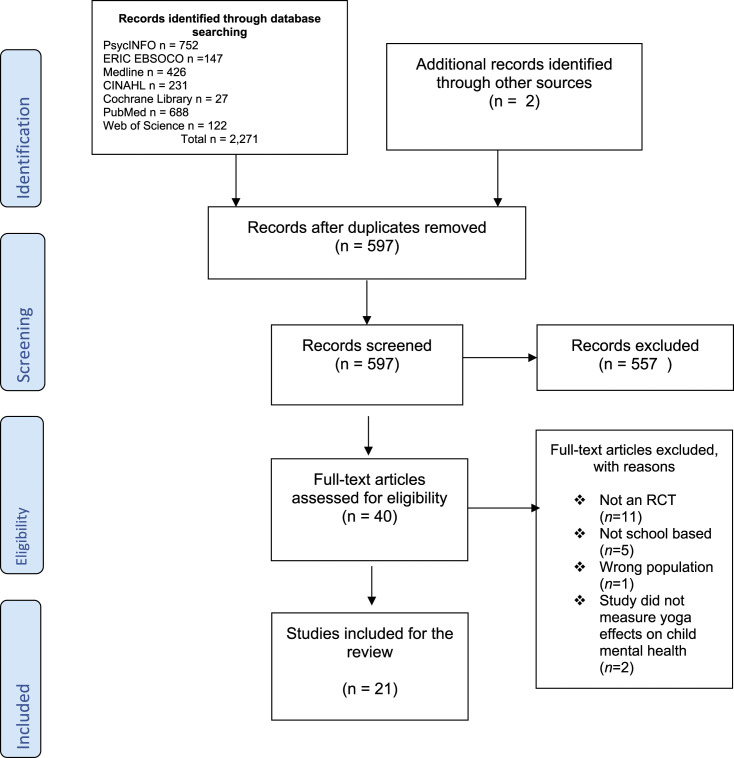
PRISMA 2009 Flow Diagram.

Forty potential results remained, of which a further 19 were excluded following applying the set-out inclusion and exclusion criteria in Table 1; finally, 21 papers remained. Figure 1 summarises the selection process of the included papers.

**Table 1.** Inclusion and exclusion criteria.

Eligibility Criteria	Inclusion	Exclusion
Intervention	Yoga practice that includes postures and meditation evaluating mental health (anxiety, low mood, depression) in children	Not using yoga as an intervention or mindfulness type only intervention
Study Design	Randomised controlled trials (RCTs)	Prospective or retrospective Cohorts, pilots or feasibility studies, mixed method studies
Setting	Mainstream school setting	Children in schools for Special Educational needs (SEN)
Population	Children aged 5 years old and under 16	Children aged under 5 and over 16 years
Outcome	Mental health outcomes using validated clinical assessments or parent/child/teacher self-report.’	No mental health-related outcomes reported
Publication Date	January 2008 onwards	
Language	English	Not in English
Publication status	Peer-reviewed papers	Unpublished studies such as records of ongoing research, grey literature and theses

Studies that included SEN children or where yoga was practised in school but did not measure mental health outcomes were excluded from the review. Studies were also excluded if they were carried out on children aged 17 or older, as the rationale for the review was to summarise the effects of yoga on mandatory school-aged children (5–16 years).

### Data extraction

Literature search results were uploaded to EndNoteX9, which served as a screening record against the selection criteria and was also used to remove duplicate results. Those not relating to yoga and schoolchildren were excluded from the literature review. 2271 records were screened, and 597 papers were further perused. The title and abstract of 597 papers were screened and obtained full-text journals for all papers that appeared to meet the inclusion criteria. Forty potential results remained, of which a further 19 were excluded following applying the set-out inclusion and exclusion criteria. Finally, 21 papers were included in the review. Figure 1 summarises the selection process of the included papers.

### Quality assessment

Version 2 of the Cochrane risk-of-bias tool for randomised control trials (Sterne et al., 2019) was used to summarise the results of the selected studies. Risk of bias was assessed against the four domains in the Cochrane risk-of-bias tool: Randomisation process, risk of bias due to deviations from the intended interventions, Missing outcome data, risk of bias in the measurement of the outcome and risk of bias in the selection of the reported result. Each criterion was categorised as ‘low’, ‘high’ or ‘some concerns’ for each RCT study and presented in Table 2.

**Table 2.** Systematic review included studies and data extracted from randomised control trials.

Author, Year City & Country	Sample	Intervention & Taxonomy	Control group	Duration	Evaluation	Outcome	Findings	Risk of bias
Anusuya et al., 2021 Tamilnadu India	14 – 16 years *n =* 30 yoga *n =* 30 control (Shavasana supine rest) *n* = 30 males *n* = 30 females Total: 60	Mind Sound resonance technique (MSRT) a yoga-based relaxation technique that uses mantra (A, U, M, Om, and Maha-Mrityunjaya Mantra/Chanting) to generate resonance, which is Used to induce deep relaxation	Routine activities, along with supine rest (SR), for a similar duration of MSRT.	30 minutes a day. 6 days a week for 2 weeks	Participants were assessed with state trait anxiety inventory - short form, Mind Wandering (MQW) A questionnaire, state mindfulness attention awareness Scale, and Trail-making task at baseline and post-intervention.	Evaluate a yoga-based relaxation technique’s efficacy as an extracurricular activity on the psychological state (stress, anxiety) and cognitive function.	The study results revealed a significant reduction in anxiety and mind wandering with an increased state of mindfulness, awareness, attention, and working memory in school children.	Some concerns
Bazzano et al., 2018 New Orleans, USA	8 – 9 years*n* = 20 Yoga *n* = 32 control Males = NI Females = NI Total:52	Yoga education an evidence-based urriculum for utilising yoga in the classroom	32 students, received care as usual – limited information.	40 minutes a day, 400 total minutes over 8 weeks	The brief Multidimensional students’ life Satisfaction Scale-Peabody treatment Progress Battery and the Pediatric quality of life Inventory (PedsQL) student surveys post, mid intervention.	Improve the well-being of students by incorporating dual yoga and mindfulness programming into the school, measuring stress, self-esteem, depression and anxiety.	Students who received the intervention demonstrated significantly improved psychosocial and emotional quality of life compared with their peers who received standard care.	Some concerns
Bhardwaj & Agrawal 2013 Haridwar India	10 –12 years *n =* 22 yoga *n =* 22 control group *n* = 26 males *n* =18 females Total: 44	Pranayama – breathing Bhastrika - rapid & forceful inhalation & exhalation Anulom-vilom Alternate nostril breathing Bhramri - Bumblebee Breath Various asanas – postures	Control group received no intervention.	35 minutes one month 6 days a week (except Sunday)	Indian adaptation of Battle’s self-esteem inventory for children (self-reported questionnaires) – post & pre-intervention	Develop an intervention plan based on yoga for pre-adolescent school children to enhance their self-esteem, body image and stress reduction.	The yoga group showed a significant increase in the level of total self-esteem, general self-esteem & social self-esteem.	Some concerns
Butzer et al., 2017 New YorkUSA	12 years *n =* 117 yoga *n =* 94 Control PE class *n* = 77 males *n* = 132 females Total: 209	Kripalu -meditation & breathwork Postures Sun salutations Alom vilom - Alternate nostril breathing Relaxation	P.E. as usual – no yoga was practised by control group	35 minutes twice a week for 6 months	Yoga Evaluation Questionnaire (YEQ) created by the authors Qualitative questionnaires, quantitative interviews – post-intervention & six weeks after intervention	To test the efficacy of yoga for stress management, self-esteem, emotional regulation, self-appreciation, confidence and substance misuse	Direct comparisons between the yoga intervention and PE, students' perceptions of the actual yoga lessons were mixed. Yoga may have a number of beneficial effects on factors such as relaxation, stress, mood, self-regulation, social interaction, sleep, substance use, and academic performance. Females in the study tended to experience significant long-term benefits from participating in yoga more than males.	Some concerns
Conboy et al., 2013 Western Massachusetts USA	9 – 15 years *n =* 47 yoga *n =* 25 control PE Male = NI Female = NI Total: 72	Kripalu -meditation & breathwork postures, breathing exercises, deep relaxation & meditation techniques	Regular scheduled P.E. class - no yoga was practised by control group	30 minutes 2-3 times a week for 12 weeks	Evaluation questionnaire created by the authors self-reported questionnaires qualitative interviews post & pre-intervention	Psychosocial well-being & coping/self-regulatory skills, including mood, stress, anxiety, emotion regulation, mindfulness, self-esteem, anger & positive psychology.	Students reported individual benefits, improved academic & athletic performance social benefits, such as reduced reactivity to stressful social situations. Findings support yoga’s potential to improve mental health at the individual level.	High
Daly et al.*,* (2015) New York USA	15 – 16-year-olds (one 17-year-old) *n* = 19 PE control *n* = 19 yoga *n* = 23 male *n* = 14 female Total: 38	Yoga postures, Breathing, relaxation Guided meditation	P.E. classes involved football and baseball, walking and running, relays, and other socially focused activities.	40 minutes 3 times a week for 16 weeks	Emotion regulation Index for children and adolescents (ERICA) a 16-item self-report measure completed by the child. Mindful attention awareness Scale, Self-compassion Scale, Multidimensional Assessment of Interoceptive Awareness	Examine the effects of yoga on a group of adolescents' emotion regulation & to determine if mindful awareness, self-compassion & body awareness contributed to positive changes	Findings suggested that yoga significantly affects the emotion regulation capacities of adolescents that are not present in P.E. alone. There was a low response rate for teachers’ questionnaires.	High
Fishbein et al., 2016 New York USA	9 – 12 years *n =* 45 yoga *n =* 40 control regular class Male = NI Female = NI Total: 85	Hatha yoga: postures, breathing exercises, deep relaxation & meditation techniques Vinyasa - linking all the yoga poses together	Control group received no intervention. The school did not offer regular P.E. classes.	20 sessions – 3 times a week for 50 minutes	Questionnaire Multi-rater (student, teacher), multi-method (survey, cognitive, psychophysiological) data collected before & after the yoga curriculum.	To examine if yoga practice will reduce substance use (alcohol, marijuana, illicit drugs), & improve cognitive behaviour & lead to improved psychophysiological responses to stress, anger and mood are reflected in indicators such as respiratory sinus arrhythmia, heart rate, and skin conductance.	Students who received the yoga intervention, as compared to the control condition, reported decreased alcohol use; were rated by their teachers as having significant increase in social skills; and exhibited a non-significant increase in sustained attention during a stressful task. Positive outcomes, such social skills, were improved by yoga. Increases in prosocial behaviours, such as complementing others, encouraging others, offering assistance, and attempting to bring out the best in people, were seen in the intervention group.	High
Frank et al., 2017 California USA	9^th^, 10^th^, 11^th^ & 12^th^-grade students *n* = NI yoga intervention *n* = NI business- as-usual control *n* = 81 male *n* = 74 female Total: 159	Transformative life skills (TLS), a yoga and mindful awareness-based program - postures, breathing exercises, meditation	Business-as-usual with no information about the control	30 minutes per day, 3–4 times per week for 12 weeks	Attitudes toward violence Scale, positive and negative affect schedule for children (PANAS-C) - a 27-item self-report scale, Responses to stress Questionnaire (RSQ). The somatic complaints’ subscale, Self-reported questionnaires	Examine the acceptability & effectiveness of a yoga & mindful awareness-based program on at-risk adolescent students emotional distress & prosocial behaviour.	Significant improvements were seen in school engagement & concurrent reductions in unexcused absences & detentions. Findings suggest promise for yoga-based programs to enhance adolescent emotion regulation & serve as a protective factor against the onset & progression of mental health. Sustained duration or increased dosage may be required to impact more general indices of overall mood & affect significantly.	High
Gaurav et al., 2013 Pune India	16 years *n* =30 yoga *n* =30 control business as usual Male only Total: 60	Asanas – Postures Sarvangasana – Shoulder stand Halasana – Plough Pose Bhujangasana - Cobra Pose Shalabhasana - Locust pose Dhanurasana - Bow pose Ardha-Matsyendrasana - Half Lord of the Fishes pose Paschimatanasana - Seated Forward Bend Mayurasana – peacock pose Shavasana - Corpse Pose Yoga Mudra - h& gestures Bhastrika - rapid & forceful inhalation & exhalation Kapalabhati – breath of fire Pranayama – breathing	Control group participated in daily school schedule, there was no information on what business as usual was.	6 days a week, 1 hour in the morning & 1 hour in the evening except for Sundays & public holidays for 6 weeks.	Student examination anxiety scale – self-scored test questionnaires. The total number of items on this scale were 48, divided into three categories, i.e. Anxiety, Depression & stress.	The effect of yoga training on examination anxiety, depression & academic stress among students appearing for board examination.	Significant difference between the control and experimental groups in almost all the variables. Yoga practices significantly improved overall psychological parameters. Yoga training may help to control pre-examination anxiety, depression & academic stress & may improve exam results. A repeated study on students appearing for different competitive examinations was further suggested.	Some concerns
Haden et al.,2014 New York USA	11 years *n =* 15 yoga *n =* 15 control PE class Male = NI Female = NI Total: 30	Ashtanga-informed yoga practice - breathing postures, mediation Vinyasa flow - linking all the poses together for one breath Savasana - body awareness	P.E group did soccer and volleyball, indoor walking program to encourage moderate levels of physical activity	3 times a week, 30 minutes per session for 12 weeks	Positive and negative affect Scales [PANAS] for children, Child behaviour Check List [CBCL] completed by parents. Self-perception Profile for children [SPPC], Warwick-Edinburgh mental well-being Scale (WEMWBS) Self-reported questionnaires for both parents & children. Home practice was encouraged.	Determine the effects of yoga on children’s emotional & behavioural Functioning, stress, anger, depression, somatic complaints, anxiety & self-esteem when compared with P.E. classes	Yoga & P.E. did not significantly impact middle school children’s functioning, possibly due to inadequate sample size, an inadequate dose of the intervention & failure to measure mindfulness & body awareness & failure to gain enthusiasm from school personnel.	Some concerns
Hagins & Rundle, 2016 New York USA	9^th^, 10, and 11^th^- grade students Mean age 5 years *n =* 48 yoga *n =* 64 control PE class Male = NI Female = 54 Total: 112	mindfulness & yoga-based exercises	P.E. class included weightlifting, stationary biking, jumping jacks, push-ups, and games such as soccer and volleyball.	40 minutes twice a week over a total of 58 classes. Every 4 weeks, a thematic unit introduced across the academic year	Response to stress questionnaire (RSQ), teacher and student versions of the behaviour Rating Inventory of Executive function (BRIEF), Self-reported questionnaires measured stress, emotional regulation & executive function.	Examine the effects of a year-long school-based yoga program on academic performance & explore potential mediating effects of emotional regulation & stress.	Students assigned to yoga classes had a significantly higher GPA. In focus groups at the end of the year, yoga students expressed improvements in self-regulation & decreases in mental stress. Authors suggested that if yoga genuinely alters self-regulation, studies should optimally measure not only self-report but also observational behavioural measures & physiological measures (e.g., response to stress, heart rate variability).	Some concerns
Hagins et al., 2013 New York USA	10 years *n* = 15 yoga 10 male & 5 Female *n* = 16 control PE 7 male & 8 Female Total: 31	Breathing Asana – posture Vinyasa - linking all the poses together Savasana - body scan	yoga and P.E. classes occurred for the same duration and frequency and at the same time of day throughout the study. PE class included games such as soccer and volleyball.	50 minutes per session – 3 times a week for 15 weeks	The mental arithmetic task (MAT) measuring stress reactivity in children: Heart rate & Blood pressure post-intervention Self-reported questionnaires from children & parents	Measure if a yoga program can significantly reduce stress reactivity compared to P.E. class & explore the feasibility of implementing behavioural stressors in a New York city public school.	15 weeks of the yoga program did not provide significant differences in stress reactivity compared to P.E. control group.	Some concerns
Halliwell et al., 2018 South West England, United Kingdom	9 – 11 years *n =* 190 yoga *n =* 154 control PE class Total: 344 girls, no data on boys	Warrior pose Downdog, Crab, Cobra, Updog, Plank, Locust, Dolphin, Boat, Eagle, Tree, Forward Folds, Chair, Airplane, Bridge, breathing exercise humming, Relaxation	The control group attended P.E. lessons as usual.	40 minutes weekly for 4 weeks	The Appearance subscale of the body esteem Scale for children, the body Surveillance subscale of the Objectified body consciousness Scale-youth, body Appreciation Scale-2 for children, the positive and negative affect Scale for children	Impact of a 4-week yoga intervention on pre-adolescents' body image and mood. All participants completed the postintervention questionnaires 1 week after the final yoga session and follow-up questionnaires 6 weeks later.	Significant improvements in body image and mood across the yoga and the P.E. control group. While P.E. was conceptualised as a placebo control condition, it may have been an intervention, prompting positive effects on body image and mood. Yoga delivered once a week for 4 weeks may not be sufficient to afford benefits over P.E.	High
Khalsa et al., 2012 oston USA	15 – 19 years mean age = 16.8 *n =* 74 yoga *n =* 47 control PE class *n* = 70 male *n* = 51 male Total: 121	Kripalu yoga - postures, breathing, relaxation, meditation, awareness	P.E. classes-as-usual group - no treatment control	30–40 minutes 2-3 sessions per week over 11 weeks	The self-report of Personality (SRP) version of the behaviour Assessment Survey for children Version 2 (BASC-2) for children, the resilience Scale (RS), the perceived stress Scale (PSS), the Inventory of positive psychological Attitudes-32R (IPPA). Self-report questionnaires measuring mood, anxiety, perceived stress, resilience & other mental health variables	The benefits of yoga on mental health, anger management & stress	Yoga participants showed statistically significant differences over time compared to control group on measures of anger control and fatigue/inertia. Yoga interventions are feasible in a school setting within the school curriculum. Generally, positive qualitative feedback suggested that the yoga intervention was perceived as an acceptable practice by parents and students in the school setting.	High
Mendelson et al., 2010 Baltimore USA	10 – 15 years *n =* 51 yoga *n =* 46 control wait list Male = NI Female = 59 Total: 97	Asanas - postures Breathing meditation	Control group received no intervention.	45 minutes 4 days per week for 12 weeks	Depressive symptoms the short mood and feelings Questionnaire—child Version, positive and negative emotions the emotion Profile Inventory (EP), Relations with peers and school people in My life (PIML), the Responses to stress Questionnaire – focus groups with children and teachers	To evaluate the feasibility & acceptability of yoga intervention & to assess its promise for improving stress, cognitive & emotional regulation	Yoga group reported significant improvements on problematic physiological & cognitive patterns of response to stress among youth. Yoga showed promise in implementation in urban public schools.	High
Noggle et al., 2012 Western Massachusetts USA	16 – 17 years*n* = 36 yoga *n* = 15 PE control Male = NI Female = 61% Total: 52	Kripalu yoga - postures, breathing, relaxation, meditation, awareness	P.E.-as-usual group (learning the history, rules, tournament, tennis, volleyball, hockey, football, frisbee, and baseball, ropes course, backcountry living skills, stress management first aid and planned parenthood health and wellness. Yoga was not included	30 minutes 2-3 sessions per week for 10 weeks	The Profile of mood States-short form (POMS-SF), he positive and negative affect schedule for children (PANAS-C), the 10-item perceived stress Scale (PSS), the Inventory of positive psychological Attitudes-32R (IPPA), the 25-item resilience Scale (RS), the State-Trait anger Expression Inventory-2™ (STAXI-2), the Child Acceptance and mindfulness measure (CAMM) Yoga evaluation questionnaire at the end of the study to students in the yoga group.	Evaluate preventive efficacy for psychosocial well-being. Yoga would improve overall well-being such as stress, mood, and anxiety by both decreasing negative aspects & increasing positive aspects.	Yoga group showed statistically significant improvements in anger control, resilience, and fatigue/inertia. No changes were observed in positive affect, perceived stress, positive psychological traits, resilience, or anger expression. Both positive outcomes (& lack of outcomes) were tentative & required replication in larger, more definitive trials.	Some concerns
Pandit & Satish, 2014 Tamil Nadu India	9 – 12 years *n =* 46 yoga *n =* 32 Health training group *n =* 27 control waitlist Male = NI female = NI Total: 105	Asanas – Postures Surya namaskar – Sun salutation Breathing Meditation	Health training group was a non-yogic intervention, limited information on the health training and a control waitlist.	12 sessions over 6 months	Self awareness and emotional regulation Scale, Raven’s programme matrices test cancellation test, Questionnaires, self-awareness & regular scales,	Differences between the 3 groups- the yoga intervention group, the non-yogic intervention group & the time-lagged group on the anthropometric, cognitive, personality factors & self-awareness & self-reported behavioural regulation over both short term (3 months) & long term (6 months) period on anxiety and anger.	Significant improvement in yoga intervention group compared to non-yogic intervention and time lagged comparison group. Effects of yoga were not seen until 3 months later. It does not work if children are busy with other activities at school. The study presented a strong case for utilising yoga as a health intervention embedded in the curriculum. They recommended that yoga research with children should focus on long-term intervention studies, and yoga may be used in many contexts.	High
Quach et al., 2016 Ohio USA	12 – 17 years *n =* 68 yoga *n =* 61 mindfulness *n =* 57 control waitlist P.E *n* = Female 62% *n* = male 38% Total: 198	Hatha yoga: postures, breathing exercises, relaxation	The waitlist control group attended regular P.E. classes, they did not receive the yoga or mindfulness intervention.	45 minutes twice a week for 4 weeks	The perceived stress Scale 10, the screen for Child anxiety and related emotional Disorders (SCARED), the Child Acceptance and mindfulness measure (CAMM) Questionnaires -Daily home practice via CD/Audio monitored through a written home practice log - collected once a week.	Examine the effectiveness of mindfulness meditation & hatha yoga on perceived stress, anxiety & anger control.	Significant increase in working memory capacity for participants in the meditation group. Participants in the mindfulness meditation condition showed significant improvements in memory, whereas those in the hatha yoga and waitlist control groups did not. No statistically significant between-group differences were found for stress or anxiety.	Some concerns
Telles et al., 2013 Haridwar India	8 – 12 years *n* = 49 yoga *n* = 49 PE *n* = 68 male *n* = 38 Female Total: 108	Asana – postures Pranayama – breathing	P.E group jogging, Relay races/games, Spinal twisting, Bending sideways, rapid bending forwards & backwards.	45 minutes a day, 5 days a week for 3 months	Eurofit bathing 10 items Teacher rating performance	To compare the effects of yoga with P.E. on physical fitness, cognitive functions, obedience, social skills and self-esteem	Both groups showed significant improvements in a test for physical fitness. Social self-esteem improved after P.E. compared to yoga. These are possible effects of two interventions, with a degree of uncertainty due to the absence of a third control group.	Some concerns
Velásquez et al., 2015 Bogotá, Columbia	10 – 15 years *n =* 68 yoga *n =* 57 control *n* = 40 male *n* = 55 Female Total: 125	Satyananda yoga – integrate all aspects of the individual: Physical, energetic, mental, emotional, psychic & spiritual Asanas – postures Pranayama – breathing. Yog Nidra – relaxation/meditation	The control group received no intervention	2 hr sessions (Unknown per week) over 12 weeks 24 sessions in total	Self-reported questionnaires (Strengths & Difficulties questionnaire) focus group meetings asking open-ended questions at the end of the intervention.	The efficacy of a yoga programme implemented in a low-socioeconomic status school for the prevention of depression, anxiety, & aggression.	Yoga intervention showed statistically significant differences when compared to PE classes. Yoga may be beneficial in decreasing depression problems, especially for elementary school students and boys. The study relied on student feedback only and not from the teachers.	High
White 2012 Boston USA	9 – 11 years *n =* 70 yoga *n =* 85 waitlist control Girls only Total: 125	Yoga-based mindfulness, Asanas – Postures	The control group participants were offered yoga classes after the completion of the experimental group.	2.5 hours weekly class for 8 weeks 45 minutes of homework guided by a compact disk	self-assessed questionnaires - the Feel Bad Scale, the school-agers coping Strategies Inventory, the Global self-worth subscale of the self-perception Profile for children, the Healthy self-regulation subscale of the mindful Thinking and Action Scale for adolescents,	Assess if yoga reduces perceived stress, enhances coping abilities, self-esteem & self-regulation & explore the relationship between the dose of the intervention & outcomes.	No significant differences between groups were found. The intervention group reported higher perceived stress scores & greater frequency of coping – bias may be due to self-assessment questionnaires.	High

Abbreviations: Mind wandering questionnaire (MWQ) is a questionnaire tool used by the researchers to measure low mood and depression, n = number, NI = No Information, P.E. = Physical Education.

### Narrative synthesis

A meta-analysis was not possible due to the heterogeneity of the studies, as the outcome measures varied across all studies and not all studies examined anxiety or depression as an outcome. The findings of studies on children with common mental health problems are presented in Table 2. Where studies reported comparable findings on mental health, potential similarities in the intervention were provided, including duration and timing of the intervention were also summarised.

The findings of studies measuring expected mental health outcomes were presented, and findings were synthesised according to the trial comparator group. For example, yoga intervention in the control group was hypothesised to improve mental health outcomes. The findings were grouped by those researchers reporting consistent findings, such as improvements or no change. Where studies reported consistent findings, similarities between them in terms of setting, nature of intervention and outcome measures were examined. Due to the small number of RCTs included in the review high risk of bias from synthesis was not excluded.

## Results

### Characteristics of included studies

The search strategy identified 597 potential outputs, of which 21 were included in the review. Each stage of the inclusion process is shown in the PRISMA flow diagram in Figure 1. Most of the RCTs were carried out in the USA (n = 14), India (n = 5), Columbia (n = 1) and the UK (n = 1).

The trials' sample size ranged from 30 (Haden et al., 2014) to 344 participants (Halliwell et al., 2018). The yoga interventions in all 20 studies were heterogeneous, from the duration to the frequency of yoga sessions. These ranged from 30 minutes daily over five weeks to a once-a-week session over a year. The included studies are summarised in Table 2.

All the trials examined a face-to-face yoga intervention in the school setting. All the trials recruited students from the school they were attending. Most trials compared yoga intervention to an active comparator like P.E. (12 trials) but gave basic information about the P.E classes. Where information was available, the authors have included this in an extra column about the control group in Table 2. None of the control groups received yoga intervention except for White (2012), however the control group participants in their study were offered yoga classes after the completion of the experimental group. There was limited information regarding when the intervention was offered. Five trials compared yoga to a waitlist comparator, and four trials compared yoga to a regular class comparator. Only two trials had two comparator groups to a yoga intervention (Pandit & Satish, 2014; Quach et al., 2016). These trials are reported separately in the narrative synthesis.

### Common mental health problems

Sixteen trials investigated the effects of practising yoga on stress, thirteen on anxiety and five trials on self-esteem (Bhardwaj and Agrawal, 2013; Butzer et al., 2017; Conboy et al., 2013; Telles et al., 2013; White, 2012), twelve trials on depression symptoms (mood) (Bazzano et al., 2018; Butzer et al., 2017; Conboy et al., 2013; Fishbein et al., 2016; Frank et al., 2017; Gaurav et al., 2013; Haden et al., 2014; Halliwell et al., 2018; Khalsa et al., 2012; Mendelson et al., 2010; Noggle et al., 2012; Velásquez et al., 2015), ten trials on anger/self-regulation and five trials on body awareness (Bhardwaj and Agrawal, 2013; Butzer et al., 2017; Daly et al., 2015; Halliwell et al., 2018; Khalsa et al., 2012). Ten trials were classified as having a high risk of bias, and twelve trials with some concerns. No trial scored low for risk of bias.

### Narrative synthesis of yoga intervention

One trial examined Mind Sound Resonance Technique (MSRT), which involved closing the eyes and experiencing internal vibrations and resonance developed whilst chanting the syllables A, U, M, Om, and Maha-Mrityunjaya Mantra for 30 minutes. In contrast, the control group performed supine rest (S.R.) for the same duration (Anusuya et al., 2021).

(Pandit and Satish, 2014) trial had two comparators. The first was the health training group that, practised simple exercises that mimicked the yoga intervention, comprising 5 minutes of jogging, deep breathing and relaxation, and the second was a waitlist. Quach et al., (2016) trial compared yoga intervention to mindfulness control and a waitlist control group.

All the included studies had at least one form of pranayama (breathing) and several asanas (yoga postures). Although not all the studies where clear what type of pranayama was used. Four trials used Kripalu yoga (Butzer et al., 2017; Conboy et al., 2013; Khalsa et al., 2012; Noggle et al., 2012); Bazzano et al. (2018) used Yoga Education Ashtanga Vinyasa (Bazzano et al., 2018), two trials used Hatha Yoga (Fishbein et al., 2016; Quach et al., 2016), Velásquez et al., (2015) used the Satyananda Yoga tradition. Frank et al. (2017) used Transformative Life Skills (TLS), a yoga and mindful awareness-based program. Haden et al. (2014) used Ashtanga-informed yoga practice that consisted of physical postures, breathing practices, and relaxation techniques, including short meditation practices and class rules that reflected yoga’s moral and ethical components. Nine trials incorporated a manualised yoga-based intervention that contained both a combination of asanas and pranayama (Bhardwaj & Agrawal, 2013; Daly et al., 2015; Gaurav et al., 2013; Hagins & Randle, 2016; Hagins et al., 2013, Halliwell et al., 2018, Mendelson et al., 2010, Pandit & Satish 2014; White, 2012).

Sixteen trials evaluated yoga intervention using self-reported quantitative questionnaires with the school children. One trial used qualitative interviews (Conboy et al., 2013), three studies included teacher questionnaires (Daly et al., 2015; Fishbein et al., 2016; Telles et al., 2013), two studies included parent questionnaires (Haden et al., 2014; Hagins et al., 2013), one study used focus groups with the children at the end of the intervention (Velásquez et al., 2015).

### Narrative synthesis Stress

Fourteen trials reported a reduction in stress following yoga practice, and four trials found no reduction in stress (Haden et al., 2014; Hagins et al., 2013; Hagins and Rundle, 2016; White, 2010). Both Haden et al. and White observed increased rates of stress in the yoga group compared to the P.E. classes.

### Anxiety and Anger

Eleven trials saw a reduction in anxiety following yoga, whereas three saw no reduction in stress following yoga intervention (Haden et al., 2014; Khalsa et al., 2012; White, 2010). Five trials reported a reduction in anger following yoga practice (Conboy et al., 2013; Fishbein et al., 2016; Khalsa et al., 2012; Pandit & Satish, 2014; Velásquez et al., 2015); however, Haden et al. found no difference in anger.

### Depression (Mood)

Nine out of twenty-one trials saw a reduction in depressive symptoms such as mood. However, Haden et al., Bazzano et al., Bhardwaj et al., Butzer et al., Conboy et al., and Telles et al. did not find any significant reduction in depressive symptoms.

### Self-esteem

Haden et al., Bazzano et al., Bhardwaj et al., Butzer et al., Conboy et al., and Telles et al. found that the practice of yoga increased self-esteem. While, White et al. found no significant difference in self-esteem but found yoga practice provided positive coping abilities.

### Other symptoms

Butzer et al., Daly et al., Hagins & Rundle et al. found positive results in emotional regulation in children following yoga. Bhardwaj et al., Daly et al., Hanwell et al., Khalsa et al., Pandit & Satish found positive results in body awareness in children practising yoga. Conboy et al. and Dally found that children had more self-compassion in the yoga group. Conboy et al. also reported that children in the yoga group presented with healthier behaviours. Haden et al. trial found no statistical significance between yoga and the P.E. group regarding social problems, somatic complaints, and emotional and behavioural function, whereas Telles et al. found positive results in academic performance, social skills and child obedience in the yoga group.

## Discussion

This is the first systematic review to have included only RCTs that focus solely on the effects of school-based yoga intervention on mental health outcomes. Previous systematic reviews have focused on a broader range of outcome measures and included a broad range of trials, making it difficult to compare the current findings with the previous literature reviews.

This review has highlighted a need for U.K.-based RCTs as most studies were in the USA or India. The studies were also limited to small sample sizes (Bhardwaj & Agrawal 2013; Daly et al. 2015; Haden et al. 2014; Hagins et al. 2013) ranging from 30 – 44 participants and possibly the reason for the negative outcomes; for example, none of these trials were representative. There was much heterogeneity in all trial participants. Few studies collected the socioeconomic backgrounds of participants; if this information was collected, it might have been beneficial in determining the social backgrounds of families, highlighting any deprivation.

Most trials compared yoga intervention to an active comparator like P.E. but gave only basic information about the comparator, and therefore the authors included as much detail as possible regarding the control group in Table 2.

While all the studies used similar yoga interventions, none used identical evaluation measures, perhaps contributing to the different findings (Conboy et al. 2013; Noggle et al. 2012; Haden et al.2014). However, most included studies suggested the positive effects of yoga in schools. Although some quantitative studies found little statistical significance between yoga compared to the control P.E. group (Haden et al., 2014; Hagins et al., 2013), other studies found that post-yoga intervention had a negative effect (Haden et al., 2014) and increased perceived stress (White, 2012).

The findings of Conboy et al. (2013) qualitative study on yoga in schools were similar to that of (Butzer et al. 2017), Hagins & Rundle (2016), Hagins et al. (2013), Mendelson et al. (2010), and Noggle et al. (2012). However, during qualitative interviews in Conboy’s study, the students who enjoyed the active nature of P.E. said they disliked having yoga. Like Conboy et al. (2013), and Butzer et al.’s (2017) RCT, yoga was offered to 7th-grade students in the U.S. They recruited 149 students randomly assigned to participate in a 32-session yoga intervention in place of their P.E. class or regular P.E. class. Students in both groups completed pre- and post-intervention questionnaires assessing. Students reported mixed feelings about yoga enjoyment and missing P.E. class, similar to Conboy et al. (2013) and preferred to choose between yoga and P.E. and noted that athletic male students had negative opinions about replacing P.E. at school for an extended period. Therefore, when conducting such intervention studies, it may be essential to consider participant expectations at the onset of the study.

Although they found that students made positive reference to breathing techniques; however, they did not include school staff interviews, which could have provided student and school staff perceptions of the yoga intervention. Future studies could consider this to create a complete body of research. Conboy et al. (2013) reported no reduction in stress in the qualitative interviews, but positive effects reported by the children in the focus groups. This may be due to peer pressure and children needing to say positive things about the intervention.

Findings from White (2012) and Haden et al., (2014) U.S. trials revealed no significant differences between the yoga and P.E. control group. Hagins et al. (2013) trial yoga participants in their study were not too pleased doing yoga when it was apparent that some students assigned to a P.E. class had more active sports. These feelings may have influenced the outcome of their study. Therefore, future studies should consider the mechanics of RCTs and manage student expectations early on during the study design stage.

Although both groups in White’s trial reported greater self-esteem and self-regulation, the control group reported higher perceived stress scores. White believed that the adverse outcomes of the research might have been due to the incorrect use of psychometric tools and that participant awareness of stress may have facilitated coping skills. The increased awareness of stress may have resulted in more stress. In contrast, Hagins et al. (2013) stated that when students were submitted to stressor tasks in their yoga intervention, it did not reduce stress reactivity more than in the P.E. classes.

The uncertainty in trials may be due to methodological and statistical problems. Inadequate sample sizes, some variability in the types of yoga taught, short duration of yoga sessions, and inappropriate yoga postures for children may have contributed to the non-statistical significance of the results. However, these results differ from Khalsa et al. (2012), who suggested yoga can improve anger control and mood (Noggle et al., 2012) and emotional regulation (Daly et al., 2015).

Quach et al. (2016) and Pandit and Satish (2014) were the only trials to have three comparators. Pandit and Satish (2014) recruited 178 school children in 2 city schools in India and were randomly assigned to 3 conditions; 1. systematic yoga intervention, 2. non-yogic intervention, and 3. time-lagged comparison group. On the other hand, Quach et al. compared yoga practice to mindfulness and had a third waitlist group. They found significant reductions in both the yoga and mindfulness control groups.

Only two studies completed focus groups (Mendelson et al., 2010; Conboy et al., 2013). Mendelson et al. (2010) conducted three focus groups with three to seven intervention children. They found that children had positive yoga experiences and reported learning new skills that helped them daily. They were the only trial to conduct a focus group with teachers to evaluate children’s behaviour changes. Most teachers supported using yoga in class. The yoga intervention also had absences. The teacher focus group revealed that some teachers had prevented students from attending the intervention classes to punish poor behaviour in class. This might have had a negative impact on the results of this trial. If interventions are to work, schoolteachers must know the importance of the children attending the intervention.

On the other hand, Velásquez et al. (2015) trial heavily relied on students’ reports of their own and their peers’ behaviour. They did not use teachers' and parents’ evaluations because they feared it would have been biased. However, they recommended that future research use observational data to complement the students’ view of yoga practice.

The evidence regarding yoga interventions in children shows promise; however, it has methodological limitations, including small samples and little detail regarding the intervention. In some studies where the yoga practice was beneficial for the students, the study sessions were short or condensed and lasted 30 minutes (Anusuya et al., 2021; Conboy et al., 2013; Frank et al., 2017; Haden et al., 2014; Noggle et al., 2012). The recommended time for yoga is 45 minutes to 1 hour (Pandit & Satish, 2014). Almost all the studies included comparing yoga practice’s effects to P.E., and almost all observed similar results. Using different intervention groups with a different control group might be beneficial.

Future studies should investigate the long-term effects of yoga on self-esteem in children from different populations from different socioeconomic statuses and include the same protocol on large populations with follow-ups. Researchers should incorporate more waves of data collection and evaluate if the positive effects of yoga remain after the intervention has ended. As highlighted by Pandit and Satish (2014), yoga interventions do not work if initiated when children are caught up in many other academic activities. Within the developmental spans, the timing of the intervention is a crucial factor, for example, during exam time or the beginning of the academic year/end of the academic year. As (Pandit and Satish, 2014) found the effects practising yoga do not emerge until after three months of a yoga intervention. Therefore, future research should consider this and provide yoga interventions for more than 12 weeks.

Overall, the included studies have demonstrated promising results regarding yoga enhancing mental health among children and adolescents in the school setting. The review highlighted the effectiveness of yoga in helping school-aged children cope with the challenges of mental health disorders. The benefits of yoga can be disseminated to a larger population. Children will not need to depend on medical intervention when they experience stress, as they will be equipped with coping mechanisms.

### Limitations of the review

Due to the heterogeneity in the studies identified for inclusion, a meta-analysis was not possible. In addition, due to the limited information regarding yoga intervention, pranayama and asana could not be looked at separately, and not all the articles explained the intervention in full.

## Conclusion

This review has examined whether school-based yoga effectively promotes mental health in school-aged children by analysing 21 peer-reviewed RCT trials. All the studies measured mental health in children following interventions. Even though every study has validated the use of yoga in schools, there is still some ambiguity because there are so few high-quality RCTs available, and some of the results of the study were contradictory.

This review has highlighted the positive effects of school-based yoga interventions on children’s mental health. However, future research needs to be standardised, incorporate participants' wishes, and consider the views of parents and teachers, for example, the type of yoga suitable for children. Given the growing rates of child mental health, there is an urgent need for systematic examination and evaluation of yoga interventions that promote mental health.
